# Complete Genome Sequence of Spiroplasma alleghenense PLHS-1^T^ (ATCC 51752), a Bacterium Isolated from Scorpion Fly (*Panorpa helena*)

**DOI:** 10.1128/MRA.00317-19

**Published:** 2019-04-25

**Authors:** Lin Chou, Ting-Yi Lee, Yi-Ming Tsai, Chih-Horng Kuo

**Affiliations:** a Institute of Plant and Microbial Biology, Academia Sinica, Taipei, Taiwan; University of Maryland School of Medicine

## Abstract

Spiroplasma alleghenense PLHS-1^T^ (ATCC 51752) was isolated from the gut of a scorpion fly (Panorpa helena) collected in West Virginia. The complete genome sequence of this bacterium, which consists of a single 1,336,077-bp circular chromosome, is reported in this work.

## ANNOUNCEMENT

Spiroplasma alleghenense is the representative of group XXVI within this genus of arthropod-associated bacteria (1). Strains belonging to this group were all isolated from common scorpion flies (*Panorpa helena*) collected in the Allegheny Mountain region (West Virginia). This bacterium could be observed in the blood and gut fluids of adult flies. However, no apparent pathogenicity to its insect host has been reported. To facilitate future research on this bacterium, and to improve the availability of Spiroplasma sp. genomes for comparative analysis (2), we determined the complete genome sequence of the type strain, PLHS-1. This strain was triply cloned from the primary culture in M1D liquid medium (1).

The procedures for sample preparation and genome sequencing were based on those described in our previous studies (3–8). Default parameters were used for all software unless otherwise specified. The strain was acquired from the American Type Culture Collection (catalog number ATCC 51752) and grown in M1D liquid medium prior to DNA extraction using the Wizard Genomic DNA purification kit (Promega, USA). The 16S rRNA gene was amplified by PCR using the 8F/1492R primer set, followed by Sanger sequencing to verify that it matched the reference record (GenBank accession number AY189125). The shotgun sequencing utilized two Illumina libraries. The paired-end library was prepared using the Kapa library preparation kit (Roche, USA) and sequenced using an Illumina HiSeq 2000 instrument (211 + 126 bp; ∼1.7 million pairs; ∼400-fold coverage). The mate pair library was prepared using the Nextera mate pair sample preparation kit (Illumina, USA) and sequenced using an Illumina MiSeq instrument (2 × 300 bp; ∼1.5 million pairs; ∼600-fold coverage). The raw reads were trimmed from the 5′ end at the first position with a quality score of <20. *De novo* assembly using ALLPATHS-LG v52188 (9) produced 10 contigs, which were arranged into one scaffold based on mate pair reads. We utilized an iterative process to improve the assembly until the complete genome sequence was obtained. In each iteration, all reads were mapped using BWA v0.7.17 (10). The results were programmatically checked using SAMtools v1.9 (11) and visually inspected using Integrative Genomics Viewer (IGV) v2.1.5 (12). The gaps were filled based on overhanging reads, and polymorphic sites were resolved based on the majority rule. Gene prediction was based on RNAmmer v1.2 (13), tRNAscan-SE v1.3.1 (14), and Prodigal v2.6.3 (15). Homologous genes in other *Spiroplasma* sp. genomes available in GenBank (16) were identified using OrthoMCL v1 (17) and used as the reference for annotation. Manual curation was based on the information obtained from BlastKOALA v2.1 (18) and BLASTP v2.7.1 (19) searches against the NCBI nonredundant database (16). Putative clustered regularly interspaced short palindromic repeats (CRISPRs) were identified using CRISPRFinder v2017-05-09 (20).

The complete genome sequence of Spiroplasma alleghenense PLHS-1^T^ consists of one circular chromosome that is 1,336,077 bp with 29.5% G+C content; no plasmid was found. The annotation includes two sets of 16S-23S-5S rRNA genes, 30 tRNA genes (covering all 20 amino acids), 1,146 protein-coding genes, 1 pseudogene, and 1 CRISPR locus (chromosomal positions 1065812 to 1069081) containing 49 spacers.

### Data availability.

The Illumina raw reads have been deposited at the NCBI Sequence Read Archive under the accession number PRJNA205100. The genome sequence reported in this work has been deposited at GenBank/ENA/DDBJ under the accession number CP031376.
